# Field-induced *p-n* transition in yttria-stabilized zirconia

**DOI:** 10.1038/s41598-019-54588-y

**Published:** 2019-12-06

**Authors:** Marc Jovaní, Héctor Beltrán-Mir, Eloísa Cordoncillo, Anthony R. West

**Affiliations:** 1 Departamento de Química Inorgánica y Orgánica, Universidad Jaume I, Avda. Sos Baynat s/n, Castellón, 12071 Spain; 2 Department of Materials Science & Engineering, University of Sheffield, Mappin Street, Sheffield, S1 3JD UK

**Keywords:** Materials science, Materials chemistry

## Abstract

Oxide ion conducting yttria-stabilised zirconia ceramics show the onset of electronic conduction under a small bias voltage. Compositions with a high yttria content undergo a transition from p-type to n-type behavior at voltages in the range 2.4 to 10 V, which also depends on oxygen partial pressure. Surface reactions have a direct influence on bulk electronic conductivities, with possible implications for voltage-induced flash phenomena and resistive switching.

## Introduction

The application of electric fields (*dc* and *ac*) to inorganic materials is of critical importance in the development of two recently-discovered phenomena, memristive switching in thin film devices and flash sintering of ceramics. Since Chua^1,2^ predicted theoretically the existence of a fourth basic element in an electrical circuit, named the memristor and discovery of the first practical example four decades later^3,4^, research into thin film materials that exhibit novel bias-dependent behavior has increased greatly. The memory-sensitive resistor, or memristor, exhibits electrical resistive switching in response to an applied voltage and has attracted great interest for possible memory applications in electronic circuits^5,6^. The first example consisted of coupled electronic and ionic charge transfer across a TiO_2_/TiO_2-δ_ interface under the action of a *dc* bias; the resulting conductivity change required application of a reverse bias to recover the initial state^3^. Since then, many materials with switchable and retainable resistance in both ‘ON’ and ‘OFF’ states have been studied in thin film form, including MgO^7^, ZnO^8^ and VO_2_^9,10^.

The flash sintering, FS, process was discovered by Raj *et al*.^11–13^ who showed that application of *dc* fields in the range ∼ 60–120 Vcm^−1^, without applied pressure, produced sintering of polycrystalline yttria-stabilized zirconia, YSZ, powder compacts in just a few seconds starting from a relatively low initial temperature of ∼850 °C. Sintering was accompanied by a very rapid temperature rise, non-linear increase in electrical conductivity^14^ and electroluminescence^15^, of the kind demonstrated long ago with the Nernst glower^16,17^. It required a colossal amount of diffusional mass transport to produce the ultrafast rates of sintering^14^.

FS, together with the other field-assisted sintering techniques, FAST, of Spark Plasma Sintering, SPS and microwave sintering, involves application of an electric field to a green compact during sintering^18^. It has become a rapid, cost-saving and environmentally-friendly process to produce sintered materials with potential for commercial applications. FS has been applied to many ceramics^19,20^, both oxides such as TiO_2_^21^_,_ Y_2_O_3_^22^, SrTiO_3_^23^, BaTiO_3_^24^ and ZnO^25^, carbides such as SiC^26,27^, B_4_C^28^ and several metal-like non-oxide ceramics, ZrB_2_^29^ and MoSi_2_^30^.

There is much interest, and importance, attached to understanding the mechanism(s) of flash sintering and its characteristic features of rapid temperature and conductivity rise and electroluminescence. Rapid sintering is usually regarded as the main characteristic, and use, of the FS process but in fact, it appears to be a secondary consequence since single crystal materials also exhibit the characteristics of flash but without any sintering^31^. Most authors discuss FS in terms of Joule heating during flash^32,33^; others relate FS to an oxygen deficiency which is created during application of an electric field^20,34^ or to electrochemical reduction of the material, especially at sample – electrode interfaces^20,34,35^.

The present work follows earlier studies on the effect of a *small* bias voltage, in the range 1–10 V, on the electrical properties of numerous insulating, acceptor-doped barium titanate ceramics, such as BaTi_1-x_Mg_x_O_3-x_^36^ or BaTi_1-x_Zn_x_O_3-x_^37^ and oxide ion-conducting YSZ^38,39^. All showed the onset of *p-*type conduction, which was temperature-dependent, increased with voltage and was reversible on removal of the bias. The YSZ results in particular were in sharp contrast to the widespread belief that, under strongly reducing conditions, YSZ may show the onset of *n-*type electronic conduction as a result of electron injection, linked to possible oxygen loss^40^.

We report here results on Y-rich YSZ samples that, with a small bias, show the onset of *p-*type conduction but, with increasing bias, this switches to *n-*type. The switch depends critically on the magnitudes of both the applied bias and oxygen partial pressure, *p*O_2_. The sensitivity of YSZ compositions to atmosphere and bias increases with increasing Y content^41^; for that reason, the samples used here had a high yttrium content and, with 1% of Mg dopant, an overall formula Y_0.50_Zr_0.49_Mg_0.01_O_1.74_ and were labelled YSZM01.

## Results and Discussion

Pellets fired at 1400 °C for 24 h were single phase by XRD and the pattern was indexed on a cubic unit cell of defect fluorite, space group Fm $\bar{3}\,{\rm{m}}$, without evidence of a possible pyrochlore structure. SEM/EDX showed a homogeneous distribution of yttrium and zirconium, with no evidence of impurities, and a grain size in the range 0.2–0.8 μm.

**Figure 1:**
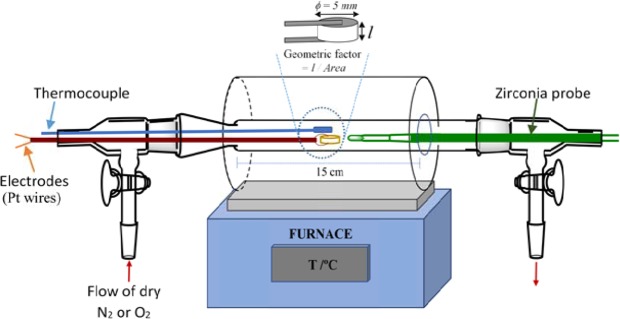
Sketch of the device used for the electrical measurements.

The experimental arrangement used for impedance measurements, with the facility to change pO_2_ and apply a *dc* bias during measurements, is shown in Fig. 1. The sample was a cylindrical pellet of typical thickness 2 mm, coated on opposite faces with hardened Pt paste electrodes and attached to Pt wires threaded through 2-hole ceramic spaghetti. The Pt wires were connected to the impedance analyser; a nominal *ac* voltage of 100 mV was used for impedance measurements, but also a small bias voltage in the range 0.5 to 20 V could be applied at the same time as impedance measurements were made. Atmosphere was a trickle flow of dried air, O_2_ or N_2_ and all sample surfaces were exposed simultaneously to the gases.

**Figure 2:**
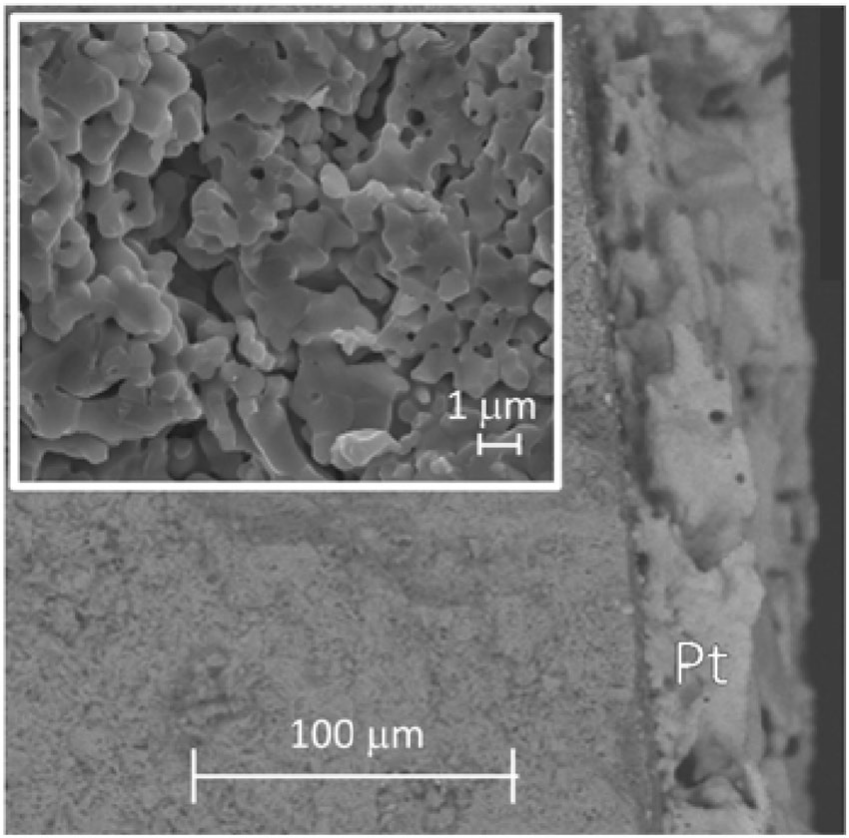
SEM of the fracture surface of a pellet with the Pt electrode attached after it was heated at 900 °C^41^.

An SEM of a fracture surface of the YSZ ceramic with attached Pt electrode of approximate thickness 50 µm is shown in Fig. 2. As discussed later, the impedance of the device consisted of two components, (i) the YSZ ceramic and (ii) the ceramic – Pt interface which was partially blocking to oxide ions but ohmic to electron transport when the ceramic was induced to show electronic conductivity.

**Figure 3:**
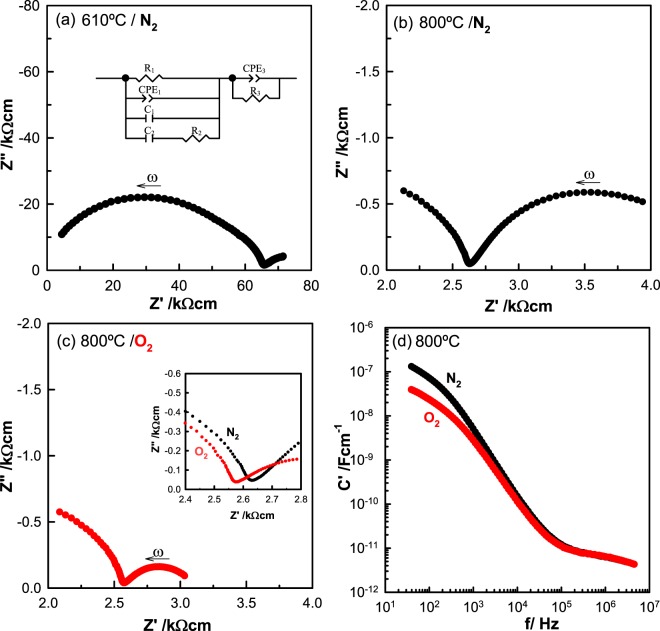
For YSZM01, impedance complex plane plots, Z*, at 610 °C (**a**) and 800 °C (**b**) measured in dry N_2_ and at 800 °C measured in dry O_2_ (**c**). C′ spectroscopic plots at 800 °C measured in the two different atmospheres (d). Inset in (**a**): Equivalent circuit used to model data.

Results are shown in Fig. 3 for impedance measurements at (a) 610 and (b) 800 °C in dry N_2_ [*p*O_2_ ∼ (1–2)x10^−4^ atm] and at (c) 800 °C in dry O_2_ [*p*O_2_ ∼1 atm]. Data at 610 °C show a broad impedance arc which, from accurate fitting to the equivalent circuit shown as the inset in (a), contains as the major component, the bulk *dc* resistance, R_1_ of the sample together with a parallel resistance, R_2_ associated with a dipole relaxation process^42^, Figs. S1–S3 and Table S1. Although the dipole component, R_2_C_2_ leads to distortion of the impedance arc, it does not contribute to the value of the sample resistance that is obtained directly from the low frequency intercept of the arc on the Z’ axis. At higher temperature, 800 °C, only the low frequency tail of this arc is seen, Fig. 3(b); nevertheless, the sample resistance is readily obtained from the intercept of the arc tail on the Z’ axis.

At lower frequencies, a second arc is seen (a-c) which is associated with the sample-electrode interface impedance and is modelled by the parallel element CPE_3_R_3_. This is shown by replotting the same data as log C’ vs log f in (d); C’ reaches values >10^−7^ Fcm^−1^ at low frequencies which are characteristic of an ionically-blocking double layer capacitance. For the sample measured in O_2_ (c), the sample resistance, R_1_ at 800 °C, ∼2.55 kΩ cm is somewhat less than in N_2_ (b), ∼2.65 kΩ cm, but notably, the low frequency impedance arc and associated resistance, R_3_ is very much smaller in O_2_ (c) than in N_2_ (b). In addition, the capacitance data (d) tend towards lower values at low frequencies in O_2_ than in N_2_.

The conclusion from these results is that the sample shows both (i) oxide ion conduction from the appearance of the low impedance frequency arc associated with high capacitance values and (ii) *p-*type electronic conduction from the decrease in both R_1_ and R_3_ with increasing *p*O_2_. With increasing *p*O_2_, oxygen molecules are absorbed by the sample, pick up electrons and dissociate to form O^2−^ ions, as shown by:

1 $$1/2{{\rm{O}}}_{2}\to {{\rm{O}}}^{2-}+2{{\rm{h}}}^{\cdot }$$

In reality, the oxygen – sample interaction may involve creation of other species such as O^−^, O_2_^−^ and O_2_^2−^, as well as O^2^^−^ ions^36,37^, but the key point is that holes must be created, as evidenced by an increase in electronic conductivity. The location of holes is believed to be underbonded oxide ions associated with the Y^3+^ dopant since there are no other species in the YSZ lattice that are able to ionise readily.

**Figure 4:**
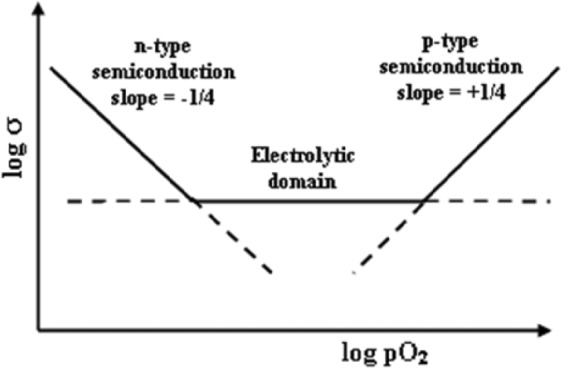
Schematic ionic and electronic conductivity domains as a function of oxygen partial pressure, pO_2_, adapted from ref. ^47^.

Under these conditions, the sample is therefore at the crossover between electrolytic and p-type domains, Fig. 4, and becomes increasingly *p*-type with increasing *p*O_2_. Many previous studies have shown that the Zr-rich YSZ compositions used as the ceramic electrolyte in solid oxide fuel cell applications are located entirely within the electrolytic domain, Fig. 4, under normal atmospheric conditions. However, there is an increasing tendency to *p-*type conduction with increasing Y content^38,41^.

**Figure 5:**
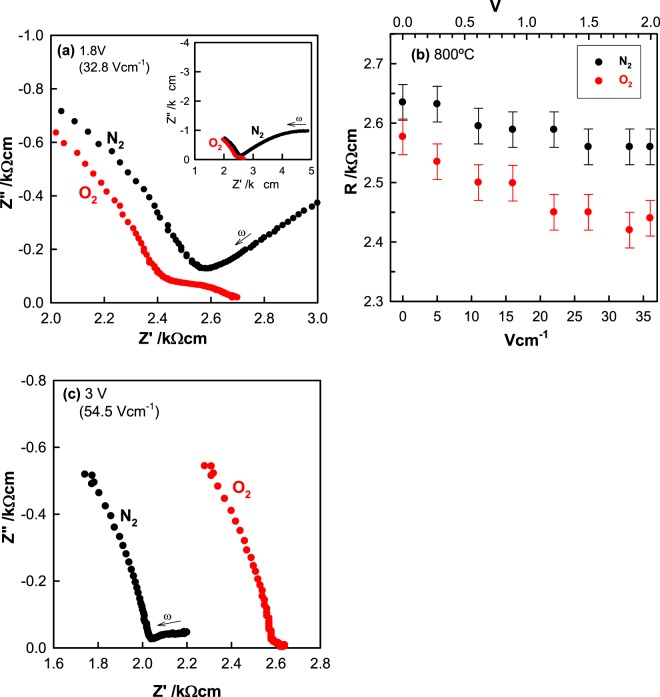
Impedance complex plane plots, Z*, for YSZM01 measured in dry N_2_ and O_2_ after applying a *dc* bias of (**a**) 1.8 V (32.8 Vcm^−1^) and (**c**) 3 V (54.5 Vcm^−1^) at 800 °C. First, measurements were done in dry N_2_ and with the *dc* voltage applied, and then, without removing the voltage, the atmosphere was change to dry O_2_. (**b**) Resistance as a function of *dc* bias voltage (less than 2 V) in the two atmospheres.

The combined effect of application of a small *dc* bias in the range 0–2 V during impedance measurements and a difference in *p*O_2_ is shown in Fig. 5(a,b). At voltages in this range, with data shown for a bias of 1.8 V as example, both the overall sample resistance and the electrode contact resistance decrease with increasing *p*O_2_, consistent with *p-*type conductivity induced by Eq. (1). Results in (b) also show that the sample resistance in either atmosphere decreases gradually with increasing bias over the range 0 to 2 V; this is attributed to an increase in hole concentration arising from reaction (1) which is preferentially driven to the right at the positive electrode. For the data shown in Fig. 5, samples were left to equilibrate for times of up to one hour to ensure steady state impedance values prior to data collection.

In contrast to the above results obtained with a bias of <2 V, impedance measurements at bias voltages of 2.4 V or greater, showed an increase in resistance of both the sample, R_1_ and electrode contact, R_3_, when the atmosphere changed from N_2_ to O_2_ and clearly suggest that conduction is now *n-*type. Therefore, a change in conduction mechanism from *p*-type to *n*-type must occur at voltages around 2.0–2.4 V. Figure 5(c) shows the impedance data for a bias of 3 V as a contrast to the data obtained at 1.8 V in (a).

**Figure 6:**
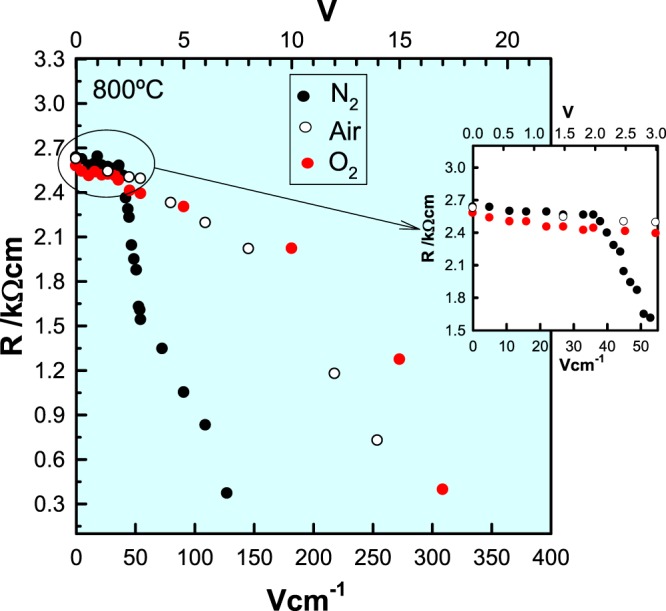
Resistance as a function of dc bias voltage at 800 °C measured in different dry atmospheres.

The effect of *dc* bias on R_1_ in three atmospheres, N_2_, air and O_2_, is summarized in Fig. 6 for a wide range of bias voltages. A more accelerated decrease in resistance was observed above 2.4 V, depending on pO_2_, but in particular, a change from *p-* to *n-*type behavior occurred, as shown by the smaller conductivities in O_2_ compared with N_2_ for a given *dc* bias. The accelerated decrease commenced at ~2 V in N_2_, but only at ~8 V in air and 10 V in O_2_. The atmosphere-dependence of this decrease and its voltage onset showed that the presence of O_2_ in the atmosphere surrounding the sample suppressed the reaction(s) responsible for the conductivity increase.

The degree of reversibility on removal of the bias depended very much on voltage. For a bias less than that which caused the *p-n* crossover, the sample impedance recovered its original value within a few minutes but at higher bias, the changes were not rapidly and fully reversed. This indicates that the initial changes on application of the bias, involving enhanced *p-*type conduction, were confined to the vicinity of the sample-electrode interfaces, but subsequently, as *n-*type behavior was initiated, more significant structural changes associated with oxygen loss occurred. We attribute this to the two-stage nature of the process by which under-bonded lattice O^2−^ ions can be released as O_2_ molecules:

2 $${{\rm{O}}}^{2-}\mathop{\to }\limits^{{\rm{step}}\,{\rm{1}}}{{\rm{O}}}^{-}+{{\rm{e}}}^{-}\mathop{\to }\limits^{{\rm{step}}\,2}1/2{{\rm{O}}}_{2}+2{{\rm{e}}}^{-}$$

In step (1), underbonded oxide ions ionize to give O^−^ ions which remain in the lattice and are the source of the holes. The released electrons are trapped by incoming or adsorbed O_2_ molecules by reactions such as Eq. (1) and:

3 $${{\rm{O}}}_{2}+{{\rm{e}}}^{-}\leftrightarrow {{{\rm{O}}}_{2}}^{-}$$

Step (1), Eq. (2), is readily reversible on removing the bias or reducing pO_2_, which drives Eqs. (1) and (3) to the left. In step (2), O_2_ molecules are released into the gas phase, the sample becomes oxygen non-stoichiometric and the rapid reversibility is lost.

**Figure 7:**
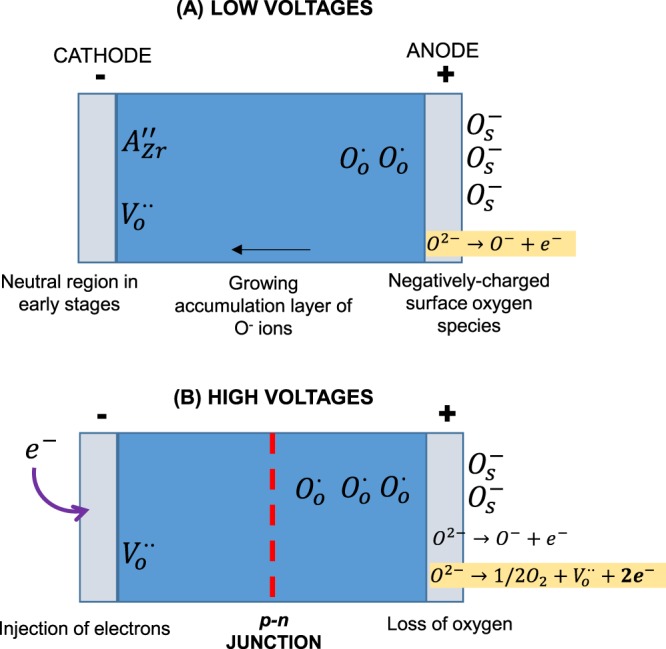
Model for voltage-dependent resistance at low (**A**) and high (**B**) voltages.

A model for this response to applied bias at low and high voltages is illustrated in Fig. 7. At low voltages, Fig. 7(A), O^−^ ions are generated at the anode which originate from a combination of lattice oxide ions and adsorbed oxygen molecules. With time, the layer containing O^−^ ions thickens from the sample surface towards the interior as more O_2_ molecules are adsorbed. We have no direct evidence for this layer thickening but note that clear evidence for it was obtained in samples of acceptor-doped BaTiO_3_ ceramics which became increasingly *p*-type with applied bias^36,37,43,44^.

At low voltages, there may be little or no O_2_ loss from the sample and the effect of the *dc* bias is to initiate capacitive charging in which the anode surface develops a net negative charge that is offset by the net positive charge of the region of the sample containing O^−^ ions^45^. Such capacitive charging also occurs spontaneously, without application of a *dc* bias, in response to an increase in *p*O_2_. Conduction of oxide ions occurs at the same time as the electronic charge separation due to capacitive charging, but appears not to be an essential part of that process. In addition, the model does not require injection of negative charges at the cathode at low voltages since this could lead to an electronic short circuit and the absence of capacitive charging.

**Figure 8:**
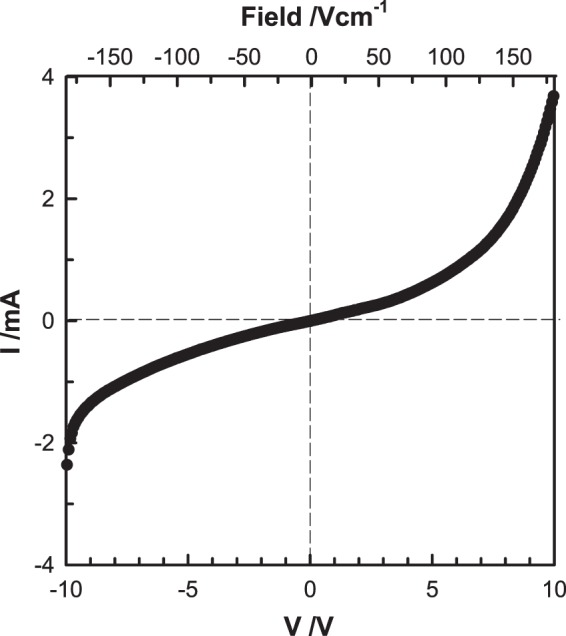
Current (I) versus voltage (V) at 800 °C for YSZM01 measured in dry N_2_ and voltages over the range −10 V to +10 V.

At higher voltages, Fig. 7(B), O_2_ gas is liberated at the anode, either by loss of O^−^ ions, Eq. (2), step (2) or by direct loss of O^2−^ ions, Eq. (1). The released electrons pass through the external circuit and are injected into the sample at the cathode. This leads to creation of, initially, a *p-i-n* junction which subsequently becomes a *p-n* junction between the *p*-type region containing O^−^ ions and the *n*-type region containing injected electrons. Evidence for the *p-n* junction is shown by the non-linear I-V plot in Fig. 8.

In conclusion, these results show that Y-rich YSZ ceramics can exist as a mixed conductor of oxide ions and either *p-* or *n-*type semi-conduction, depending on experimental conditions. At higher biases, a *p-n* junction may develop; the first step is partial oxidation of under-bonded lattice O^2−^ ions to generate a *p-*type region at the anode with mobile holes located on oxygen; the second step involves partial decomposition of the sample, evolution of oxygen gas and injection of electrons to create an *n-*type region at the cathode. We speculate that a combination of these steps may represent the first stage in the flash sintering of YSZ ceramics and also be responsible for the *p-n* junction that contributes to the luminescence that is characteristic of flash, irrespective of whether or not sintering also occurs.

It has been recognized previously that creation of electronic conductivity in YSZ may be a precursor to flash sintering^38,46^. Although various comments have been made in the literature about the possible origins of the associated luminescence, the model presented here is the first to include a mechanism for *pn* junction creation. This could then lead to luminescence associated with electron-hole recombination on passing a *dc* current through the sample.

## Methods

A composition based on the general formula Y_0.5_Zr_0.5-x_Mg_x_O_1.75-x_, with x = 0.01, labelled YSZM01, was prepared by a polymeric sol-gel procedure similar to that used in ref. ^41^. The only difference was that Mg(OOCCH_3_)_2_·4H_2_O (99%, Strem Chemicals) was added as an additional precursor to the polymeric sol-gel synthesis. The Zr alkoxide was dissolved in an ethanol-acetylacetone mixture (acacH:Zr molar ratio 4:1), the Y(OOCCH_3_)_3_·H_2_O and Mg*(*OOCCH_3_)_2_ added, the mixture stirred for 10 min and then transferred to a balloon flask and heated at 70 °C for 72 h. After formation and drying of a transparent gel the sample was given a final heat treatment at 1400 °C for 12 h. Powder samples were cooled, crushed, pressed into pellets of 5 mm diameter at 1 ton by uniaxial pressing, reheated at 1400 °C for 24 h and quenched onto Cu plate. Pellet densities for this composition were ~90%.

The samples were characterized by powder X-ray diffraction (XRD) and scanning electron microscopy (SEM) equipped with EDX; Impedance measurements were also carried out as described previously^41^. In order to avoid any effect of water and therefore, possible proton conduction, impedance data were recorded in dry atmospheres. Current (I) versus voltage (V) measurements were carried out using a Keithley Source Meter Model 2410 (Madrid, Spain)^36,37,41,43^.

## Supplementary information

Supplementary Information

## Data Availability

The data supporting the results of this study are available within the article and its Supplementary Information file.
