# Supplementary material for: Field-induced p-n transition in yttria-stabilized zirconia
Source: Sci Rep. 2019 Dec 6;9:18538. doi: 10.1038/s41598-019-54588-y (PMC6898179; doi:10.1038/s41598-019-54588-y)
Supplement: Supplementary file 1 — Supplementary Information [file 41598_2019_54588_MOESM1_ESM.pdf]

Field-induced *p-n* transition in yttria-stabilized zirconia

Marc Jovaní, Héctor Beltrán-Mir, Eloísa Cordoncillo and Anthony R West

Supplementary information

Determination of the most appropriate equivalent circuit to model and analyse impedance data is an essential step to achieving a correct physical interpretation of the data. Impedance data, Z*, of samples measured under two different atmospheres (dry N_2 and O_2) and at different temperatures were analysed using equivalent circuits consisting of appropriate combinations of resistances, R, constant phase elements, CPE and capacitances, C.

Based on previous studies of modelling similar data[S1], Fig. S1 shows the experimental data and fitted simulations for impedance complex plane plots (a and c) and C' vs frequency (b and d), with the impedance residuals (inset of b and d) and the equivalent circuit used to model data (e), for the sample measured in dry N_2 at two temperatures. From comparison of the fits to a range of circuits, it was clear that the addition of a CPE for the bulk component (R_1-CPE_1-C_1) in the equivalent circuit was necessary to fit data. The observed small separation in peak maximum frequencies of Z" and M" peaks, not shown, is a direct consequence of the presence of CPE_1 in the equivalent circuit.[S2]

C' data show two dispersions at high and low frequency with some evidence for both a limiting high frequency plateau at ~2 $pFcm^{-1}$ and a poorly-resolved intermediate frequency plateau at ~10 $pFcm^{-1}$. The high frequency plateau is attributed to the bulk capacitance. The capacitance value for the intermediate plateau is smaller than expected for a grain boundary or surface layer[S3] and instead [S1], represents an additional element in

the equivalent circuit which is an R and C in series (C_2R_2). This element represents hopping of oxygen vacancies within the dipoles, and therefore, leads to dipole reorientation but not long-range vacancy migration. The dipole reorientation is only an *ac* process but occurs at the same time as, and in parallel with, long-range *dc* conduction; therefore, R_2 does not contribute to the total resistance R_T (=R_1).

Finally, a plateau associated with the sample-electrode contact impedance, best seen with increasing temperature and at lower frequencies, (d), is included in the equivalent circuit (e). The interfacial impedance became finite, as shown in (c) and an extrapolated low frequency intercept on the real Z' axis of the impedance complex plane plot was made at high temperatures and low frequencies. Element CPE_3 that represents the sample-electrode interface was therefore modified by the addition of a parallel resistance, R_3.
The quality of fits of experimental data to the equivalent circuit can also be seen from plots of the residuals as a function of frequency. The residuals of Z' and Z" are very small over the entire frequency range indicating a good fit of the data.
Arrhenius plots of σ_T, σ_1 and σ_2 obtain from fittings to circuit Fig S1(e) of the sample measured in dry N_2 are shown in Fig S2. These data show approximately linear behaviour with very similar activation energies, 1.47 and 1.56 eV, for σ_1 and σ_2 respectively. σ_T and σ_1 have the same activation energy since σ_T does not contain a contribution from the dielectric resistance, R_2; since R_2 makes no contribution to the intercept values of R_T in impedance complex plane plots it would not be detected by standard impedance complex plane analysis.
Similar fitting was observed for the same sample measured in dry O_2, Fig S3. The sample resistance is somewhat less than in N_2 and the low frequency impedance arc is much smaller. Therefore, the sample, which shows both oxide ion conduction and *p*-type

electronic conduction becomes increasingly *p*-type in oxygen. In order to model the effect of pO_2, and the introduction of additional *p*-type electronic conduction, the equivalent circuit shown in Fig S3(c) was tested. A new resistor, R_4, was connected in parallel with R_1-CPE_1-C_1 of the bulk and the series R_2-C_2 dipole element to simulate the additional electronic conduction. The value for R_4 was obtained from the reduction in sample resistance that occurred on changing pO_2. In order to fit the data, it was assumed that R_1 was unaffected by pO_2 and its value was fixed to be the same as that at 800° C in N_2. This equivalent circuit fitted the impedance data well, especially at low frequencies. Table S1 summarizes data obtained at two different temperatures and includes the value of R_4 obtained at 800°C.

[S1] Vendrell, X. and West, A.R., Electrical Properties of Yttria-Stabilized Zirconia, YSZ Single Crystal: Local AC and Long Range DC Conduction. *J. Electrochem. Soc.*, **165**, F966-F975 (2018).

[S2] Bruce, P., West, A.R. and Almond, D. A new analysis of ac conductivity data in single crystal β-alumina, *Solid State Ionics* **7**, 57-60 (1982).

[S3] Irvine, J., Sinclair, D.C. and West, A.R. Electroceramics: characterization by impedance spectroscopy, *Adv. Mater.* **2**, 132-138 (1990).

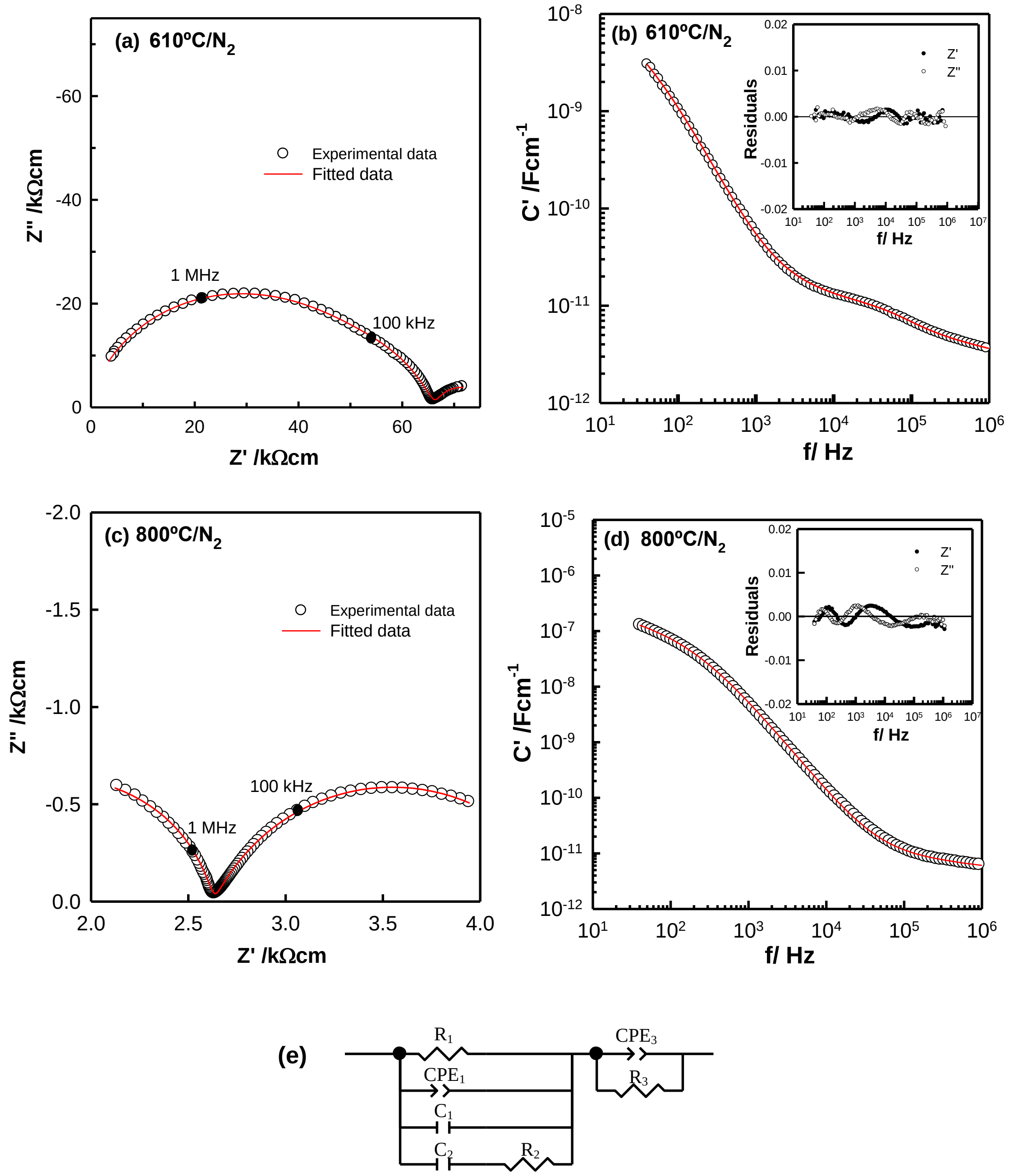

Fig S1. Experimental data and fits to the equivalent circuit (e) for impedance complex plane plots, Z*, and C' spectroscopic plots with impedance residuals (inset) measured at 610ºC (a, b) and at 800ºC (c, d). Measurements were made in dry N_2.

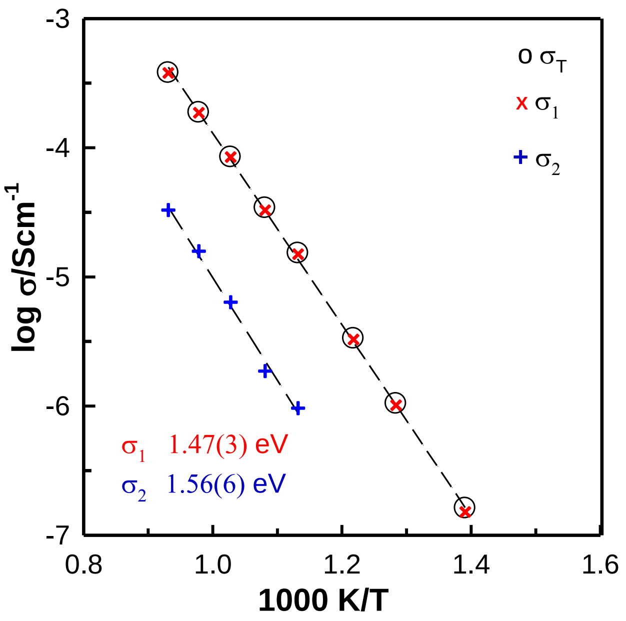

Fig S2. Arrhenius plots for total conductivity, σ_T, and for fitted values of σ_1 and σ_2 for YSZM01 sample measured in dry N_2.

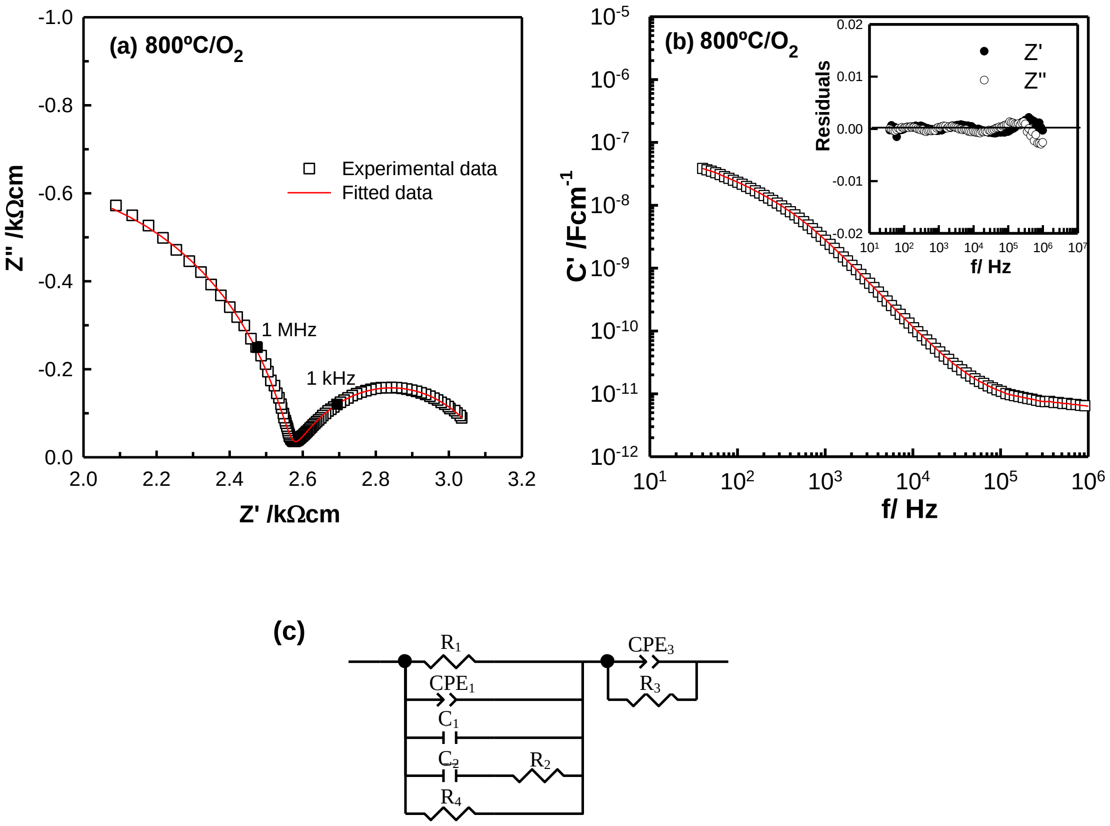

Fig S3. Experimental data and fits to the equivalent circuit (c) for impedance complex plane plots, Z*, (a) and C' spectroscopic plot (b) with impedance residuals (inset) measured at 800ºC. Measurements were made in dry O_2.

Table S1. Fitted parameters for impedance data collected at two different temperatures and atmospheres.

**T(ºC)_atm**	**R_1 (kΩcm)**	**C_1 (pFcm^{-1})**	**A_1 (pScm^{-1}rad^{-n})**	**n_1**	**R_2 (kΩcm)**	**C_2 (pFcm^{-1})**	**A_3 (μScm^{-1}rad^{-n})**	**n_3**	**R_3 (kΩcm)**	**R_4 (kΩcm)**
**610ºC_N_2**	65.8(52)	1.035(2)	187.0(2)	0.732(4)	752(3)	2.865(2)	2.03(1)	0.686(6)	13.09(14)	-
**800ºC_N_2**	2.637(1)	1.035(2)	517.5(2)	0.674(3)	11.68(2)	2.37(3)	5.14(1)	0.725(2)	1.834(4)	-
**800ºC_O_2**	2.637	1.035	517.5	0.674	10.96(9)	2.37	11.04(4)	0.676(3)	0.535(1)	111.4(5)
